# Pioglitazone Phases and Metabolic Effects in Nanoparticle-Treated Cells Analyzed via Rapid Visualization of FLIM Images

**DOI:** 10.3390/molecules29092137

**Published:** 2024-05-04

**Authors:** Biagio Todaro, Luca Pesce, Francesco Cardarelli, Stefano Luin

**Affiliations:** 1 NEST Laboratory, Scuola Normale Superiore, Piazza San Silvestro 12, 56127 Pisa, Italy; luca.pesce1@sns.it (L.P.); francesco.cardarelli@sns.it (F.C.); 2 NEST Laboratory, Istituto Nanoscienze-CNR, Piazza San Silvestro 12, 56127 Pisa, Italy

**Keywords:** fluorescence lifetime imaging microscopy (FLIM), pioglitazone characterization, insulinoma (INS-1) cells, PLGA nanoparticles, phasor-FLIM analysis, MATLAB tool, drug delivery

## Abstract

Fluorescence lifetime imaging microscopy (FLIM) has proven to be a useful method for analyzing various aspects of material science and biology, like the supramolecular organization of (slightly) fluorescent compounds or the metabolic activity in non-labeled cells; in particular, FLIM phasor analysis (phasor-FLIM) has the potential for an intuitive representation of complex fluorescence decays and therefore of the analyzed properties. Here we present and make available tools to fully exploit this potential, in particular by coding via hue, saturation, and intensity the phasor positions and their weights both in the phasor plot and in the microscope image. We apply these tools to analyze FLIM data acquired via two-photon microscopy to visualize: (i) different phases of the drug pioglitazone (PGZ) in solutions and/or crystals, (ii) the position in the phasor plot of non-labelled poly(lactic-co-glycolic acid) (PLGA) nanoparticles (NPs), and (iii) the effect of PGZ or PGZ-containing NPs on the metabolism of insulinoma (INS-1 E) model cells. PGZ is recognized for its efficacy in addressing insulin resistance and hyperglycemia in type 2 diabetes mellitus, and polymeric nanoparticles offer versatile platforms for drug delivery due to their biocompatibility and controlled release kinetics. This study lays the foundation for a better understanding via phasor-FLIM of the organization and effects of drugs, in particular, PGZ, within NPs, aiming at better control of encapsulation and pharmacokinetics, and potentially at novel anti-diabetics theragnostic nanotools.

## 1. Introduction

Fluorescence lifetime analysis (FLA) has demonstrated significant potential for non-invasive, real-time analysis across a diverse range of compounds and materials. Specifically within biomedicine, it has been applied in several key areas, like (i) measurement of intracellular biochemical parameters [1,2,3,4,5,6,7], (ii) resolving the physical state of encapsulated fluorescent drugs [8,9,10,11,12,13,14,15,16,17,18]; and (iii) biomedical diagnostics and neuroscience research [19,20,21,22,23,24,25,26,27].

Fluorescence lifetime imaging microscopy (FLIM), unlike traditional fluorescence microscopy where the contrast is given by emission intensity, delves into the time dimension of fluorescence decay. In the simplest implementation, the contrast is given by fluorescence lifetime, the average time a fluorophore remains in the excited state before emitting a photon. This temporal information can unveil valuable insights into the microenvironment, molecular interactions, and biochemical properties of fluorophores since fluorophores exhibit characteristic fluorescence decay patterns influenced by their surroundings. These decay patterns provide a quantitative means to assess molecular processes, such as energy transfer, molecular binding, and changes in local pH. In particular, FLIM has been widely used to analyze cells’ metabolic states, starting with the pioneering work of Ragan et al. (1969) focused on the oxidation–reduction changes in flavin and pyridine nucleotides in perfused rat liver [28]. Lakowicz et al. (1992) conducted work on imaging the short-lived fluorescence of both free and protein-bound NADH, determining the changes in their ratio, a critical parameter for assessing cellular metabolism [29]. Ferri et al. (2020) recently exploited this parameter for investigating the metabolic response of Insulinoma 1E cells to glucose stimulation, and Azzarello et al. (2022) used it to examine the metabolic response of α and β cells to glucose in living human Langerhans islets [30,31].

However, the average lifetime is not always enough to characterize the decay patterns; one can study the complete decay curves, fitting them with multiexponential decays, in order to dissect various contributions in each pixel of an image. However, fit parameters often have too big uncertainties, especially if the single component lifetimes are too close (closer than a factor of 2 or 3) and if the measurements are noisy, as it happens especially in the case of fast measurements. Moreover, each component is characterized by an amplitude and a lifetime, and all these parameters are not always easy to represent in an image. A valid alternative for representing FLIM data is given by the phasor approach (phasor-FLIM): this involves transforming fluorescence decay data into points on a phasor plot, essentially a two-dimensional representation in the complex plane of the Fourier transform of the decay curve at a fixed frequency, with real component G on the x-axis and imaginary component S on the y-axis. Each pixel in a FLIM “image” is mapped to a position in the phasor plot, creating clusters of data points that represent various fluorescence decay characteristics. In the case of a monoexponential decay, expected for a simple pure compound in solution, the phasor falls along the universal semicircle (centered on (1/2, 1/2) and with radius 1/2, for positive S), with shorter lifetimes more on the right; for bi- or multi-exponential decays, the phasor falls within this semicircle. When more than a compound is present in a pixel, the phasor falls in a position given by the average of the phasors of each compound weighted by the contribution of the compound to the total intensity of fluorescence in that pixel; in simpler terms, the phasor position in such cases is given by the linear combination of the ones of pure compounds. This aspect provides valuable and intuitive insights into the compounds composition within a pixel of an image, allowing for a more comprehensive understanding of complex molecular environments.

For example, Stringari et al. (2011) utilized the phasor approach to distinguish various metabolic states of germ cells in live tissue, providing crucial insights into cellular metabolic function [2]. A recent study by Tentori et al. (2022) demonstrated the utility of phasor-FLIM in unveiling the supramolecular organization of doxorubicin (DOX) in the standard Doxoves^®^ liposomal formulation (DOX^®^), underscoring its potential in elucidating the complex interplay between nanoparticles and biological systems. They determined that DOX^®^ includes three different fractions: crystallized DOX (98%), free DOX (1.4%), and an unexpected liposomal-membrane-bound DOX (0.7%); relative concentrations were determined through phasor-FLIM and spectroscopy on pure standards, demonstrating applicability for studying the state of encapsulated drugs from the production to within living matter [15]. A bottleneck that still affects FLIM adoption by a broader audience is the lack of a facile tool for presenting in an intuitive way the large amount of data generated, particularly for researchers without extensive expertise in data analysis [32]. There are some pieces of software developed to this aim: commercial ones such as those provided by Becker & Hickl GmbH (Berlin, Germany), PicoQuant (Berlin, Germany), or Leica Microsystems (now part of Danaher, Washington, DC, USA), and a few freely distributed ones such as SimFCS, FLUTE, PAM and one developed by some of us [33,34,35,36]. However, they are still limited in representing the whole two-dimensional phasor plane in the FLIM image, using a color scale changing mostly in one direction within the phasor plot or just highlighting in the image a subset of the phasor plot.

From a biomedical point of view, polymeric nanoparticles (NPs), and in particular poly(lactic-co-glycolic acid) (PLGA), have garnered significant interest as versatile drug delivery platforms due to their biocompatibility, controlled release kinetics, and tunable physicochemical properties [37,38,39]. Recent advancements in nanoparticle engineering, including surface modification and functionalization, offer opportunities to enhance drug encapsulation efficiency, target-specific delivery, and cellular uptake [40,41,42,43,44,45,46,47,48,49,50,51]. In particular, a drug that would benefit from an NP formulation is pioglitazone (PGZ), a thiazolidinedione derivative, which is a well-known pharmacological agent for addressing insulin resistance and hyperglycemia in type 2 diabetes mellitus (T2DM) patients. Metabolic disorders, such as T2DM, present a significant global health challenge, driving the exploration of innovative monitoring devices for diabetes management and therapeutic approaches to mitigate associated complications and improve patient outcomes [52,53]. In this context, the PGZ mechanism of action involves the activation of peroxisome proliferator-activated receptor gamma (PPARγ), a nuclear receptor pivotal in regulating glucose and lipid metabolism, adipogenesis, and inflammation. Despite PGZ’s clinical efficacy, challenges related to its pharmacokinetic properties, including low solubility and bioavailability, underscore the importance of exploring innovative drug delivery strategies to optimize therapeutic outcomes. A recent study from Todaro et al. (2022) elucidated the most reliable synthesis method for PGZ-loaded PLGA nanoparticles, highlighting the potential of PLGA as a suitable nanocarrier material and demonstrating controlled release capabilities for PGZ [54]. The nanoprecipitation method employed for these formulations has proven effective, showcasing favorable attributes such as size, polydispersity index, encapsulation efficiency, drug loading, cost-effectiveness, and processing time [54]. The successful synthesis and characterization of PGZ-loaded PLGA NPs set the stage for a deeper investigation into the drug’s intrinsic fluorescence signals. Among all the methods available for the polymeric NPs characterization [55,56,57,58,59,60,61,62], FLIM can play an important role in terms of manufacturing process control, and also explain drug pharmacokinetics properties.

Here, we present and make available a tool (coded in MATLAB) that maps the whole phasor plot to different hue and saturation values and represents the distribution in the phasor plot with an intensity that considers each pixel intensity in the image. The color map can be chosen in different ways, in order to characterize more than two possible coexisting decays characteristics of possibly different fluorescent molecules.

As a proof of concept, we apply this tool to the study of different phases of PGZ, and we analyze its effects (both when free and encapsulated within NPs) on the metabolism of INS-1E cells, an insulinoma cell line. Indeed, the investigation presented in this article exploits PGZ intrinsic fluorescence to collect information regarding its physical state through the application of FLIM, by analyzing the differences in the fluorescence decay of PGZ when existing in various states, such as dry crystalline, solvated, or dissolved forms. Furthermore, we present the observed phasor distributions for PLGA nanoparticles, empty or encapsulating PGZ. Finally, we investigate cellular NAD(P)H autofluorescence variations between its free and bound form in the presence of PGZ-loaded PLGA nanoparticles.

## 2. Results

**Figure 1:**
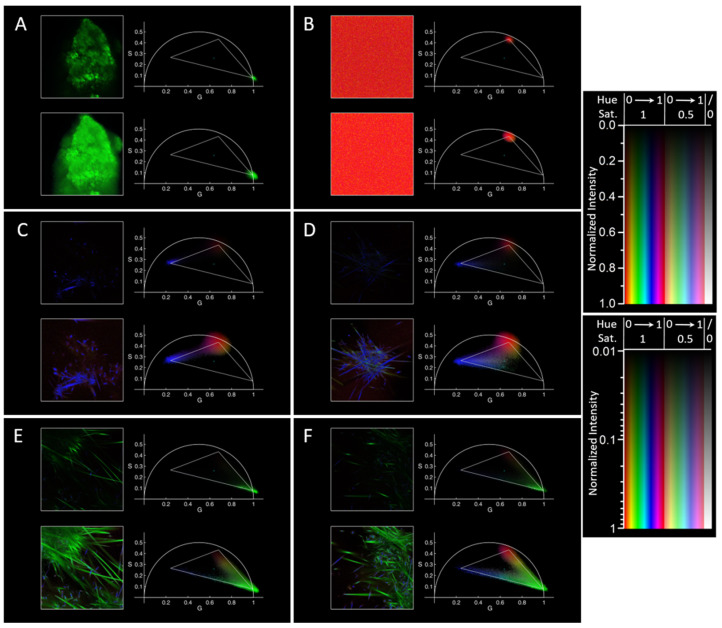
Characterization of free PGZ using FLIM. For each panel, on the left are reported the FLIM images and on the right the phasor plots (with corresponding colors): (**A**) PGZ solid, (**B**) PGZ in DMF at 37 °C at time 0, (**C**) PGZ in DMF at 37 °C at 20 min, (**D**) at 30 min, (**E**) at 50 min, and (**F**) at 55 min in a different position; in the phasor plot, vertices of the triangle correspond to the “principal” points, and the “center” point, where the saturation is 0 (see Section 3 and Section 4.5) is shown in cyan. For all the panels: on top, figures with a linear intensity scale (normalized to maximum); on the bottom, the same figures with a logarithmic intensity scale starting from a 0.01 fraction of the maximum intensity in the image; on the right these intensity scales are shown in corresponding positions for various values of hue and saturation. Note how the evaporation of DMF with time causes the formation of PGZ crystals, and that the used color coding based on the position of the phasor plot for each pixel allows appreciating at a glance the different forms of PGZ and their position within the images. The side of the square microscopy image is 106 µm here and in all the following figures.

We developed an algorithm, implemented in MATLAB R2017B (and tested in version R2023B), which allows determining at a glance the position in the phasor plot of the phasor corresponding to each pixel in the image; in the phasor plot, the phasors distribution is weighted with the intensity in each pixel, and is shown with colors corresponding to the ones in the image. The colors are determined in order to highlight the position of the phasors with respect to chosen “principal” points in the phasor plot, ideally corresponding to the position for pure fluorophores, and in particular with respect to the segments joining them. The first examples of this representation are reported in Figure 1, using the same color coding in every image, with a light gray triangle superimposed on the phasor plots that highlights the considered principal points at its vertices; the choice of these principal points will be explained in the following section. Intensities are shown in a linear but also in a logarithmic scale, in order to appreciate the shape of the observed features, but also the less intense “tails” of the phasor distribution and the less intense pixels in the image, respectively.

### 2.1. Phases of Pioglitazone (PGZ)

The data reported in Figure 1 derive from a detailed examination, employing FLIM, of free PGZ in different conditions.

Initially, solid PGZ was examined on a glass slide, revealing a monoexponential decay characterized by a very short lifetime (phasor on the universal semicircle at high S and low G; Figure 1A); to this point, we assigned a hue of 1/3, corresponding to green. In parallel with what was observed on doxorubicin [5], we consider the observed short lifetime indicative of the crystalline nature of solid PGZ; the shortness of the lifetime is most probably caused by self-quenching of PGZ molecules strongly interacting with each other in the close packing of the crystal. We then analyzed PGZ dissolved in dimethylformamide (DMF), a solvent where it is highly soluble, differently than in water (we did not observe enough fluorescence intensity from a saturated solution of PGZ in water to determine its decay characteristics with our setup). DMF-solved PGZ presents an almost monoexponential decay at a longer, intermediate lifetime (Figure 1B, time 0 from the positioning of a drop of DMF with solved PGZ on the microscope cover glass). We assigned to this phasor position a hue of 0 (and 1), corresponding to red. Notice that we also measured a pure DMF solution, finding no autofluorescence (and therefore positions in the phasor plot close to the origin of axes, as expected for noise or for light uncorrelated with the pulsed laser reaching the detector). Leaving the drop at 37 °C, the DMF started to evaporate, the PGZ reached saturation, and crystals started to grow within the solution (Figure 1C,D). Unexpectedly, the phasor position in the pixels corresponding to these observed crystals did not fall close (or tended towards) the “green” point mentioned above; instead, they tended towards a different point in the phasor plot, corresponding to a longer multiexponential lifetime, and to which we assigned a hue of 2/3 corresponding to blue. We consider this a sign of a different crystal form, a “solvated crystal”, where many DMF molecules (or possibly water impurities, since we did not use anhydrous DMF and all the experiments were performed in air, containing some humidity) enter in the composition of the crystal and influence the interactions amongst PGZ molecules. Note that in Figure 1C, with intensities represented with a linear scale, the two populations of the bright but less abundant pixels with solvated crystal and the dim but much more numerous pixels with solved PGZ are both visible in the phasor plot (while the last population would overwhelm the first one employing standard representations of the phasor plot), but this last population red color is not appreciable in the image on the left. Instead, using a logarithmic intensity scale, the “red” part of the image is evident, and the crystals can be seen in their entirety; in the phasor plot, a “smear” connecting the “blue” and “red” principal points is visible, and this corresponds to pixels where the point spread function contained both part of a crystal and part of the solution. In Figure 1D, a shift of the average phasor towards the crystalline region is observable, indicating the progression of crystallization, evident also from the higher number of bigger crystals in the image on the left. Moreover, some low-saturation points, also towards a green color, start to appear (visible especially in the figure with logarithmic intensity scale), indicative of the fact that some crystals more similar to the original ones begin to form. Subsequent analysis at 50 and 55 min revealed fluorescence arising predominantly from a crystalline sample, evidenced by a significant shift towards very short lifetimes in the phasor analysis (Figure 1E,F). These observations underscore the dynamics of PGZ dissolution and crystallization over time, allowing for the conceptualization of a triangle, with the three reference species (the solid form, the solved form, and the “solvated crystal” form of PGZ) at its vertices.

### 2.2. Phasors of PGZ-Loaded PLGA NPs

Subsequently, empty PLGA nanoparticles and PGZ-loaded PLGA NPs were synthesized and analyzed. The synthesis of these samples was carried out as previously described, with slight modifications [54]. The characterization of three independent formulations is shown in Table 1, demonstrating reproducibility over size, PDI, Z potential, and encapsulation efficiency.

**Figure 2:**
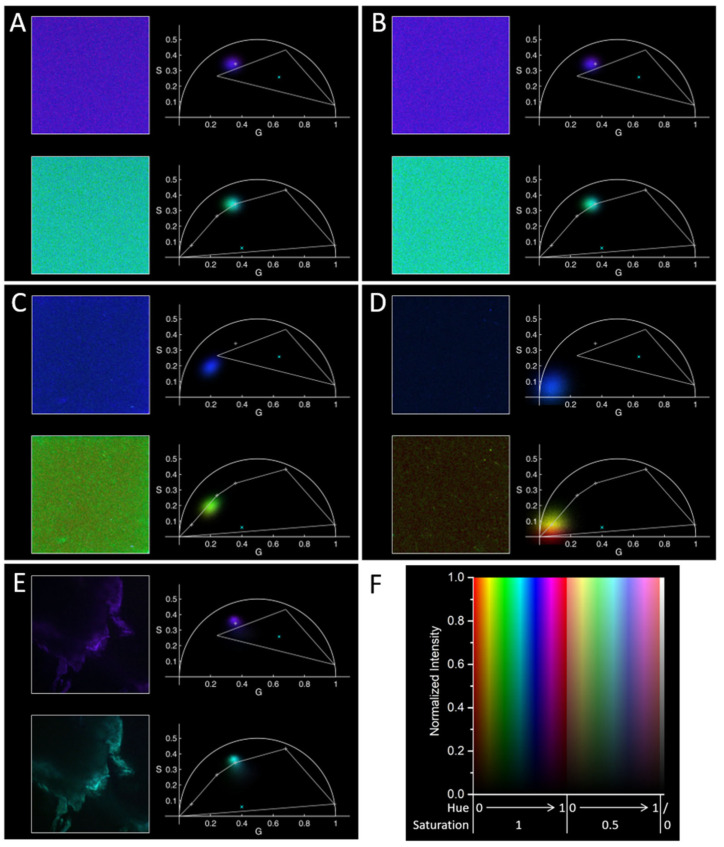
Characterization of empty PLGA nanoparticles and PGZ-loaded PLGA NPs using FLIM. For each panel, on the left are reported the FLIM images and on the right the phasor plots (with corresponding colors) for exemplary cases for NPs (**A**–**D**) and for solid PLGA (**E**): (**A**) empty PLGA NPs in RPMI, (**B**) PGZ-loaded PLGA NPs in RPMI, (**C**) empty PLGA NPs in PBS, (**D**) PGZ-loaded PLGA NPs in PBS, (**E**) solid PLGA. A linear scale was used for the intensity normalized to the maximum in each picture (like on top in Figure 1; color scale reported in panel (**F**)). Two color codes were used, one like in Figure 1 (top figures in each panel), one with additional principal points (see main text), as shown in the right bottom figure in each panel (vertices of the polygon and light gray crosses), with hue of 0/1 at (0,0) and increasing clockwise, and a “center” point (cyan ×) inside but towards the bottom of the polygon.

The samples were analyzed at the microscope using FLIM. Unfortunately, repeating the experiments on different batches we observed very different values of fluorescence intensities and phasor positions. For both types of samples, phasors fell approximately along a line connecting the origin (phasor position of noise) and a point shown in the top right graph of panels A–E of Figure 2 as a light gray cross; this line passes very close to the vertex of the triangle corresponding to the solvated phase of PGZ. Moreover, for both samples, the phasors in case of high enough fluorescence were close to the point cited above. Some examples of these measurements are reported in panels A–D of Figure 2, on top of each panel with the same color coding and linear intensity scale used for the top half of each panel in Figure 1. Being a dispersion, in the images reported on the left part we can see a quite uniform field, with some possible aggregates (possibly moving during the measurements) visible especially for the experiments with the lowest fluorescence. These behaviors did not depend on the buffer where the NPs were dispersed (we used PBS and RPMI, the last for comparison with experiments in cells reported below). In order to understand the origin of these fluorescence decay characteristics, we measured the lifetime characteristic of solid PLGA, discovering an unexpected fluorescence, whose characteristic phasor fell on the point cited above (see Figure 2E). In this case, the observed image reported on the left is not optimal, most probably because we used the standard setup, and in particular an objective with silicon oil as immersion medium, to visualize a piece of mostly transparent solid placed on a cover glass in the air; in any case, the fluorescence decay characteristics do not depend on the quality of the image.

These experiments demonstrate the possibility of measuring PLGA nanoparticles with phasor-FLIM; however, the too high noise, the autofluorescence of the used PLGA most probably overwhelming the one of the encapsulated PGZ, and the closeness of the PLGA phasor to possible ones characterizing the PGZ do not allow for understanding the state of PGZ within the PLGA. One could check if different batches of PLGA present lower autofluorescence, and try to obtain more concentrated NPs colloids, but this is going beyond the scope of this work. We wanted to explore all the possible positions for the phasors linked to fluorescence arising from our NPs and to understand if there would be some fluorescence arising from them in the measurements presented in the following section.

**Figure 3:**
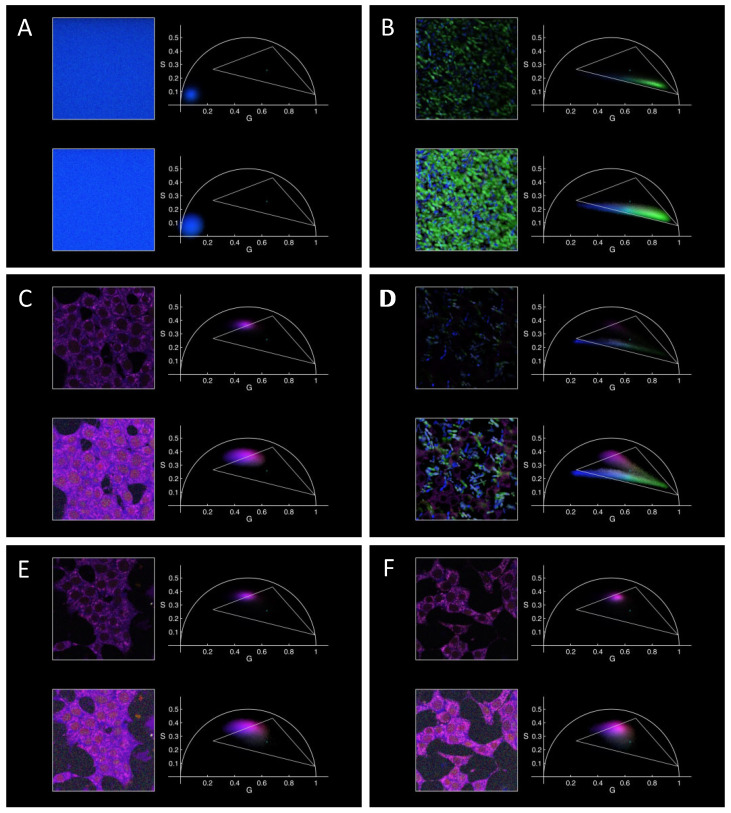
FLIM of PGZ and of INS-1E cells in RPMI medium, also upon co-incubation and incubation with nanoparticles for 24 h at 37 °C. (**A**) RPMI, (**B**) PGZ in RPMI, (**C**) INS-1E in RPMI, (**D**) INS-1E in RPMI in the presence of 0.1 mg/mL PGZ, (**E**) INS-1E in RPMI in presence of empty PLGA NPs, (**F**) INS-1E in RPMI in presence of PGZ-loaded PLGA NPs. Each panel has the same composition, color coding, and intensity scales as in Figure 1 (and in the top parts of the panels in Figure 2 for the linear intensity scale), in order to compare more easily the results and to show at-a-glance possible signals arising from the species observed in the previous cases.

In the images discussed above (top part of each panel A–E in Figure 2), all the phasors result in being blue/violet, even if they are in different positions. For the purpose of highlighting even more the potential of our algorithm in presenting phasor-FLIM data, we show in the bottom part of panels A–E of Figure 2 the same data, but using more and different “principal points” (in a clockwise order: the origin, the average phasor measured in a RPMI solution as shown in Figure 3A below, the point for solvated PGZ, and the average phasor position for solid PLGA, for dissolved PGZ, and for dry crystalline PGZ). This representation allows for easily visualizing the different observed decay characteristics for the NPs colloids. In particular, in panels A and B of Figure 2 we observe the same cyan color of PLGA, as shown in panel E. In panel C, we see a phasor distribution elongated along the line connecting the origin (or the RPMI typical phasor) with the PLGA phasor, intermediate between these points, with a green color. In panel D, we observe a phasor distribution closer to the “noise zone”, and the different red to yellowish-green colors are quite mixed in the image on the left, demonstrating that the distribution is mostly caused by noise in the measurements, with just some possible aggregates with a more yellowish-green color, more towards the PLGA/NP typical phasor.

### 2.3. Autofluorescence Characteristics Changes in INS-1E Cell in the Presence of PGZ-Loaded PLGA NPs

In the final experiments, we incubated INS-1E cell lines with the previously analyzed samples at 37 °C for 24 h and measured them in our two-photon FLIM microscope. First, we checked that the used RPMI medium was negligibly fluorescent, resulting in a phasor falling very close to the origin, the zone where noise falls (Figure 3A). As a control, 20 μL of 10 mg/mL PGZ was incubated in 1980 μL of RPMI at 37 °C (final nominal PGZ concentration of 0.1 mg/mL or ~280 µM), both alone (Figure 3B) and in the presence of cells (Figure 3C). In both cases, we observed a precipitation of PGZ, as expected for a maximum reported PGZ solubility in water around 0.07 mg/mL or 200 µM at 37 °C [63]. We observed microscopic solids characterized by a complex fluorescence decay, illustrated by a phasor distribution ranging within points very close to the ones characterizing the solvated crystal in DMF and the anhydrous crystal species (Figure 3B). The differences between this case and the one in DMF can be easily rationalized considering the different impact of included water (and not DMF) on the interactions between the PGZ molecules within the solid. The slightly curved line formed by the local maxima in the distribution points more towards the possible presence of a continuum of species with different quenching rather than just a mixture of two species [64].

The presence of precipitated crystals in Figure 3D ensures that the solved drug is at saturation, and therefore at its maximum possible concentration; we will show that it does not make it more difficult to understand the observed fluorescence decays from within the cells thanks to the way we are presenting the data. We also report the FLIM results on control cells without anything added in the medium (Figure 3C). The fluorescence of cells with the used channel characteristics (two-photon excitation at 740 nm, emission around 440 nm) arises mostly from NAD(P)H, and indeed the cell phasor positions are in agreement with previously observed ones [7]. Subsequently, the cells were incubated with PGZ-loaded nanoparticles and empty nanoparticles (as negative control), and the corresponding phasor-FLIM results are reported in Figure 3E,F with the same color coding. Again, the fluorescence from the cells is the only one noticeable, while no fluorescence arising from NPs can be identified in the phasor plot. Note that, although the cell autofluorescence was not considered in deciding the color coding used in Figure 3 (the same used in Figure 1), the phasor corresponding to cell-containing pixels fall in a zone characterized by a violet color, therefore they are clearly discernible from the PGZ precipitate in Figure 3D.

As hinted in the introduction, free and bound NAD(P)H, more abundant when oxidative metabolism is slower and faster, respectively, have different fluorescence decay characteristics and therefore different positions in the phasor plot [7,29,30,31]. By looking carefully at the data reported in Figure 3C–F, even if the color encoding is not ideal for this purpose, it seems that the negative control (Figure 3E) and the cells alone (Figure 3C) present a very similar phasor distribution, while in the case of cells in the presence of PGZ-loaded NPs, the phasor distribution is shifted from slightly at the left (Figure 3C,E) to the right (Figure 3F) of the top left side of the drawn triangle (as clearer in the top images in the panels, with the linear intensity scale). This could also be the case for the cells in the presence of free PGZ, but the analysis is hindered in such cases by the presence of the more fluorescent PGZ precipitate.

**Figure 4:**
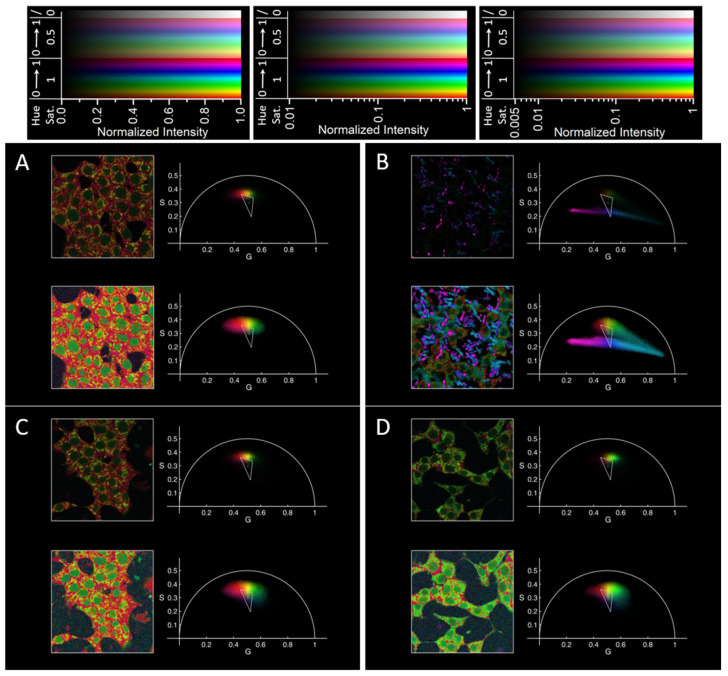
FLIM characterization of INS-1E cells interacting with PGZ and with the nanoparticles considered in this work. The same data of Figure 3C–F are here reported with a different color encoding for phasor position (principal points at the vertices of the triangle and center point in cyan in the right part of each panel), in order to better appreciate the position of phasors characterizing fluorescence arising from within the cells. (**A**) INS-1E in RPMI, (**B**) INS-1E in RPMI in the presence of PGZ, (**C**) INS-1E in RPMI in the presence of empty PLGA NPs, (**D**) INS-1E in RPMI in the presence of PGZ-loaded PLGA NPs. For each panel: on the left, FLIM images; on the right, phasor plots with corresponding colors; on the top, a linear intensity scale is used (the most on the left on the top of the figure), on the bottom a logarithmic one starting from 1% (panels (**A**,**C**,**D**), center color scale on top of the figure) or from 0.5% (panel (**B**), rightmost color scale on the top of the figure) of the maximum intensity in each image. Note that, on average, the apparent intensities of cells in the bottom image of panel (**B**) are similar to the ones on the top images in the other panels.

To facilitate grasping these fluorescence decay characteristics changes (and to show the versatility and potential of our methods for FLIM data presentation), we changed the color encoding. We considered three principal points (Figure 4, vertices of the triangles): two based on the position of the phasors for cells in RPMI only and in the presence of PGZ-loaded PLGA NPs (examples in Figure 3C,F), and a third one in the middle of the smear corresponding to the PGZ precipitate. It is again easy to discern the cells from the precipitated solids (Figure 4B); moreover, in this case, it is much easier to appreciate the average shift from a situation with the NAD(P)H phasor distribution characterizing more bound NADH (at a relatively longer lifetime, red zones in Figure 4) to one characterized by more free NADH (green in Figure 4), widely accepted to be linked to a decrease in oxidative metabolism (mitochondrial respiration) or to an increase in glycolytic metabolism [7,30,65]. Moreover, with this color coding, it is possible to check the possible heterogeneity amongst different cells (see, e.g., the redder ones, especially in Figure 4B, bottom left panel) or even the modulation of NAD(P)H status within single cells.

The shift observed in the phasor related to free and bound NAD(P)H in response to PGZ can be attributed to its impact on cellular metabolism. PGZ, a member of the thiazolidinedione class of antidiabetic drugs, exerts its effects primarily by activating peroxisome proliferator-activated receptor gamma (PPARγ), a nuclear receptor involved in the regulation of glucose and lipid metabolism. Activation of PPARγ by PGZ leads to transcriptional regulation of genes involved in insulin sensitivity, glucose uptake, and lipid metabolism. Furthermore, PGZ has been shown to enhance mitochondrial function and biogenesis, leading to increased oxidative phosphorylation and ATP production in insulin-sensitive cells, but to have an opposite effect on beta cells in pancreatic islets, from which INS-1E cells are derived and of which they are a model, with a decrease in aerobic metabolism and in insulin secretion [66,67,68]. This metabolic shift towards decreased aerobic metabolism in beta cells is reflected in changes to the cellular redox state and is therefore in agreement with our observed alterations in the free and bound NAD(P)H ratio.

## 3. Discussion and Conclusions

The applications of fluorescence-based techniques have transcended conventional limits, offering profound insights into the molecular complexities of various pharmaceutical agents. For instance, the drug organization within a polymeric or biologic matrix can be studied using phasor-FLIM approaches [11,15]; by leveraging the capabilities of FLIM, researchers can gain insights into drug release kinetics, intracellular trafficking, and metabolic responses, thereby facilitating the rational design of drug delivery systems with enhanced efficacy and safety profiles. However, the widespread adoption of FLIM in scientific, industrial, and clinical fields is hindered by the complexity of the needed instrumentation and data analysis and presentation. Some steps have been taken towards the mitigation of the first point [69], and this work represents another step towards a more intuitive presentation of the results of fluorescence lifetime microscopy, in cases where the impact of three or more species present in a sample needs to be easily recognized and mapped.

The MATLAB scripts and functions used in this manuscript (and made freely available) allow defining interactively the “principal” positions in the phasor plots, i.e., the ones considered important in the data presentation (e.g., the ones of pure species or the same species in different environments), to which particular hue values and high saturations are assigned. Moreover, it is possible to define a “center” point where the saturation becomes zero (else, it is considered to be in the middle of the principal points). It is also possible to consider these points as constant and to apply the same color coding to a big number of files containing phasor-encoded images. Importantly, the correspondence between the phasor plot position and (hue, saturation) pair is in principle bijective, so that it can be reversed. In the phasor plot, the intensity follows a distribution where each pixel in the image is weighted by its intensity so that the phasor regions with the highest contributions in the observed field can be noticed at a glance without the need to define an arbitrary threshold (even if the user can impose low and high thresholds, also interactively, if needed). More details are contained in the comments at the beginning of each file.

We applied our algorithms in order to study different phases of PGZ, visualize PLGA nanoparticles, and observe cells and the metabolic shift within them. An interesting observation, which could be neglected in studies involving the effect of non-perfectly soluble drugs on adherent cells, is the one of precipitated 0.1 mg/mL PGZ in various solid phases. This precipitation would largely increase the local concentration of the drug in the proximity of the cells and therefore can lead to an overestimation of the drug effect at the nominal concentration (probably, unreachable by the free form of the drug). Instead, we did not observe any precipitate with the empty or PGZ-loaded NPs, while we could reach the nominal concentration of 0.1 mg/mL of PGZ contained within them. Indeed, an important conclusion arising from our label-free fluorescence lifetime measurements on the NADH-linked metabolism of living cells, is that the effect of PGZ-loaded NPs is higher than the one of dissolved PGZ, even if in this last case crystals of precipitated PGZ were very close to the adherent cells. This points towards a higher effectiveness of the NP-encapsulated drug versus the free-form one.

We could not observe a clear signal from PGZ phases within NPs, probably because of an unexpected too high autofluorescence of the used batch of PLGA or a too low final equivalent concentration of PGZ. However, we believe that the results presented here prove the usefulness of our method for presenting FLIM data in complicated samples, especially when autofluorescence (of drugs, cells, or other components) can be exploited. Indeed, any material can be studied with a setup similar to the one presented here if it is fluorescent, in our case upon two-photon excitation. Depending on the fluorescence properties of other biocompatible polymers (batches) for the synthesis of NPs, they could behave as shown here, could have higher fluorescence intensity and therefore more reproducible phasors, or might be fluorescence-free and therefore make easier studying the drug organization within them, as it happens for liposomes [11,15]. Regarding the drug, if fluorescent, we expect its fluorescent decay to be different in crystalline or solved form in most if not all cases (because of the completely different environment that should change at least the fluorescence quantum yield). However, it is difficult to predict if there could be other phases with different fluorescence decays as it happens for the “solvated” PGZ crystals. In any case, the system presented here is potentially applicable to other drug-nanoparticle conjugates.

Finally, the results reported here represent a step forward in understanding the effect of PGZ on beta-type cells and in developing drug-embedded NPs with more controlled pharmacokinetics, and they can be useful to suggest different characterizations of autofluorescent materials, e.g., various drugs, and of cell response to drugs and/or to changes in their environment.

## 4. Materials and Methods

### 4.1. Materials

Pioglitazone (PGZ), Resomer^®^ RG 503 H, PLGA acid terminated (lactide:glycolide 50:50, Mw 24,000–38,000, referred to as PLGA), D-(+)-Trehalose dihydrate, 4-Morpholineethanesulfonic acid (MES), polyvinyl alcohol (87–89% hydrolyzed, Mw approx. 18,000, PVA) and all other chemicals were purchased from Merck KgaA (Darmstadt, Germany) and were used without further purification. INS-1E cells were kindly provided by Prof. C. Wollheim, University of Geneva, Medical Center [70]. Ultrapure water was generated in-house using a MilliQ plus System (Merck KgaA, Darmstadt, Germany), while all the other solvents, including acetone and dimethyl sulfoxide (DMSO), were purchased from Merck KgaA (Darmstadt, Germany). All other chemical reagents were purchased from Aldrich (Saint Quentin Fallavier, France) or Acros (Noisy-Le-Grand, France).

### 4.2. Synthesis and Characterization of PGZ-Loaded PLGA Nanoparticles

PGZ-loaded PLGA NPs were synthesized using the nanoprecipitation method, with some modifications based on our previous publication [15]. In brief, 400 μL of PLGA (24–32 kDa) dissolved in acetone (10 mg/mL) and 50 μL of PGZ dissolved in DMF (10 mg/mL) were combined and added dropwise to 1600 μL of an aqueous phase comprising 1:1 MES buffer (0.1 M, pH 6.2) and PVA (4%, 18 kDa) under continuous stirring. The resulting suspension was stirred for a minimum of 3 h to facilitate solvent evaporation and nanoparticle formation. Subsequently, the nanoparticles were collected via dialysis (1 kDa cutoff) against 2 L of 1:1 MES buffer (0.1 M, pH 6.2) and PVA (4%, 18 kDa) solution at 4 °C overnight. The physicochemical properties of NPs (including hydrodynamic radius, polydispersity and ζ-potential) were characterized using a Zetasizer Nano ZS equipment (Malvern Instruments Ltd., Worcestershire, UK) at 25 °C, while the entrapment efficiency (EE%) was characterized using a Shimadzu Nexera UHPLC, equipped with a Shimadzu SPD-M20A UV/visible detector, as already reported in our last publication [15]. Finally, the nanoparticles were stored in a 10 mg/mL trehalose solution until use.

### 4.3. INS-1E Cell Culture

Insulinoma 1E (INS-1E) cells [70] were cultured in a climate-controlled incubator maintained at 37 °C with 5% CO_2_. The cells were cultured in RPMI 1640 medium supplemented with 11.1 mmol/L D-glucose, 10 mmol/L HEPES, 2 mmol/L L-glutamine, 100 U/mL penicillin–streptomycin, 1 mmol/L sodium pyruvate, and 50 μmol/L β-mercaptoethanol. For lifetime experiments, cells were grown in sterilized microscopy-compatible dishes (IbiTreat μ-Dish 35-mm, Ibidi) until they reached 70% confluence (24–48 h).

### 4.4. FLIM Setup

Fluorescence Lifetime Imaging Microscopy (FLIM) was conducted using an Olympus (now Evident, Tokyo, Japan) FVMPE-RS microscope coupled with a two-photon Ti:sapphire laser (MaiTai HP, Newport SpectraPhysics, Santa Clara, CA, USA) operating at a repetition rate of 80 MHz. The FLIM data were acquired with the FLIMbox system for lifetime acquisition (ISS, Champaign, IL, USA). Calibration of the ISS Flimbox system was performed using the known mono-exponential lifetime decay of Fluorescein at pH  =  11.0 (4.0 ns upon two-photon excitation at 740 nm, collection range: 400–570 nm). The calibration sample consisted of a stock solution of 100 mmol/L Fluorescein in EtOH, prepared and diluted 1:1000 in NaOH water solution at pH 11.0 for each calibration measurement. Excitation was performed at a wavelength λ = 740 nm, fluorescence collection range was 400–570 nm, laser power was 0.6%, and acquisition was made by a GaAsP photomultiplier tube (PMT) detector with voltage at 850 V; image sampling was set at 512 × 512 pixels with 10 µs/pixel. For FLIM measurements of PGZ and PGZ-loaded PLGA NPs, experimental conditions included λ = 740 nm and a specific filter for the NADH metabolism range (420–460 nm). Used laser power ranged from 2.0 to 10.0%, and the voltage on the GaAsP PMT was set at 850 V, with image sampling maintained at 512 × 512 pixels, frame size: 106 × 106 μm^2^, with a pixel size of 0.207 × 0.207 μm^2^ and scanning at 10 µs/pixel.

### 4.5. Phasor Visualization

The pixel colors in the microscopy image and in the phasor plot have a hue/saturation/value encoding, with hue and saturation depending on the position of the phasor in the phasor plot, and intensity value given by the pixel intensity in the image, and by the sum of the intensities of points within a 2D bin (“pixel”) in the phasor plot. The intensities are always normalized to the maximum one (separately for the image and for the phasor plot) and are shown in a linear or in a logarithmic scale (actually, the scale is linear between 0 and x% of the maximum intensity, then it is logarithmic, with x = 1 unless specified otherwise). The phasor plot pixels have standard dimensions of 0.02 × 0.02 but can be specified otherwise.

Hue and saturation are calculated starting from a certain number of “principal” points (minimum two, better if at least three) in the phasor plots, ideally the positions in the phasor plot of the pure fluorescent species present in the sample, plus a “center” point. If not given when calling the function calculating hue and saturation, the user can choose them on a binned scatter plot for all the phasors in the image; ideally, most of the phasors should be included in the region having them as vertices, and this region should be (but does not have to be) convex. If the center point is not given, it is calculated as the average position of the principal points. The hue is calculated from the angle between the segments from the center point to the desired spot and from the center point to the principal points and is always increasing going clockwise or anticlockwise (depending on the order of the given principal points, eventually corrected to be always increasing in the chosen orientation if they are more than three). The principal points have equidistant values (with 0 and 1 both corresponding to the first principal point); the hue of a phasor with an angle included within the ones of two principal points is the average of the hues of these two points weighted with the angular distance from the opposite one. The saturation is zero on the central point and increases radially. Considering the intercept of the half line from the center point to the considered phasor with the line between two central points and with the borders of the universal semicircles, the hue increases (with a function proportional to sin(x) for x from 0 to 1) between 0 and a certain first value up to the first encountered intersection point, then linearly between this value and a second one between the two intersection points, then it tends exponentially to 1 for higher distances. The second value is fixed; the first one is corrected starting from a given value so that it is closer to the second one the closer the two intersection points are. In the plots shown in this work, these values were 0.95 and 0.85 (uncorrected value). The decay constant for the exponential function is the average of the distances from the center points to the two intersection points. The algorithm considers different increasing functions for nonstandard situations, possible, e.g., if the center point is chosen outside the universal semicircle and/or the region having the “principal points” as vertices.

Before calculating the phasors distribution and for the representation in the images, the values of intensities and of the G and S coordinates have been smoothed starting from their dependence on the pixel positions in the image. The intensities are treated with a 2D Gaussian filter with a given standard deviation (1 pixel in the cases shown here). The values for G and S are transformed in each pixel considering a weighted average with weights given by a Gaussian centered in the considered pixel with the same standard deviation used for the intensities, multiplied by the intensity values of the corresponding pixels.

OriginPro 8.0 was used for preliminary visualization of average phasor positions within the universal circle. MATLAB R2017B was employed for advanced mathematical modeling enabling the derivation of quantitative information on phasors, for calculating the color coding mapping, and preparing all images shown in this work. Additionally, Fiji, a free image-processing package, was used for preliminary spatial analysis of microscopy and FLIM data.

## Figures and Tables

**Table 1:** Size distributions measured using a Zetasizer Nano ZS equipment (Malvern Instruments Ltd., Worcestershire, UK), and encapsulation efficiency (EE) % measured using an analytical RP-HPLC (Shimadzu Nexera UHPLC, equipped with a Shimadzu SPD-M20A UV/visible detector) instrument. The hydrodynamic radius ± SEM (standard error of the mean), polydispersity PDI ± SEM, ζ potential ± SEM, and PGZ encapsulation efficiency % ± SEM are indicated. Results from n = 3 repetitions of measures on each of the N = 3 independently manufactured nanoparticle batches (total of 9 measurements).

		Size (nm)	PDI	ζ Potential (mV)	EE %
PGZ-loaded PLGA NPs	Average	143.32	0.25	−11.27	27.37
	SEM	2.61	0.02	0.21	0.67
Empty NPs	Average	131.92	0.12	−13.76	/
	SEM	3.76	0.01	2.11	/

## Data Availability

MATLAB codes for functions and scripts used to produce the images and the results presented in the manuscript are available at https://github.com/Stefano-Luin/phasor_plots (accessed on 3 April 2024), and can be used if this paper is properly cited in any obtained result. Datasets are available on request from the authors.
